# Xyloglucan remodelling enzymes and the mechanics of plant seed and fruit biology

**DOI:** 10.1093/jxb/erac020

**Published:** 2022-03-02

**Authors:** Tina Steinbrecher, Gerhard Leubner-Metzger

**Affiliations:** Department of Biological Sciences, Royal Holloway University of London, Egham, Surrey TW20 0EX, UK

**Keywords:** Biomechanics, cell wall remodelling, climate change, diaspore heteromorphism, dispersal, dormancy, fruit and seed traits, hemicellulose

## Abstract

This article comments on:

**Di Marzo M, Ebeling Viana V, Banfi C, Cassina V, Corti R, Herrera-Ubaldo H, Babolin N, Guazzotti A, Kiegle E, Gregis V, de Folter S, Sampedro J, Mantegazza F, Colombo L, Ezquer I.** 2022. Cell wall modifications by α-XYLOSIDASE1 are required for the control of seed and fruit size. Journal of Experimental Botany 73, 1499–1515.

The developmental transition from flowers to the mature diaspores (seeds or fruits) depends on cell growth and differentiation (Finch-Savage *et al.*, 2006; Balanza *et al.*, 2016). The plant cell wall is a dynamic nanoscale network for which the classical model and role of xyloglucan–cellulose tethers in wall structure and cell growth was challenged by recent results from genetics, biomechanics, and advanced imaging (Moulia, 2013; Cosgrove, 2018; B. Zhang *et al.*, 2021). Xyloglucan (XyG), the predominant hemicellulose, is composed of a β-1,4-glucan backbone that is consecutively substituted with α-1,6-linked xylosyl residues (Frankova *et al.*, 2013; Pauly *et al.*, 2016). Di Marzo *et al.* (2022) demonstrated that the MADS-box transcription factor SEEDSTICK (STK) specifically controls seed and fruit biology by α-xylosidase (XYL) mediated XyG remodelling.

Specific cell wall remodelling is decisive for generating the diversity in morphological, biomechanical, and physiological traits of dispersed diaspores during seed and fruit development (Steinbrecher *et al.*, 2017; Landrein *et al.*, 2019; Seale *et al.*, 2020; Arshad *et al.*, 2021; Huss *et al.*, 2021). It is of similar importance in the control of germination timing via dormancy, seed responses to abiotic stresses including heat (thermoinhibition), and seedling growth required for plant establishment and survival in a particular environment (Finch-Savage *et al.*, 2006; Shigeyama *et al.*, 2016; Finch-Savage *et al.*, 2017). A representative structural unit of XyG is composed of four β-1,4-linked glucose molecules (backbone) of which three have α-1,6-linked xylose side chains in *Arabidopsis thaliana* (XXXG; see Box 1 for nomenclature). The xylosyl residues are often modified with β-1,2-linked galactosyl residues which may be additionally α-1,2-linked with fucosyl residues (Box 1). A machinery of specific glycosyl transferases, transglycosidases, and hydroxylases generates the diversity in XyG structures, with XyG α-1,6-xyosyltransferases (XXTs) adding αXyl residues, and α-xylosidases (αXYLs) cleaving xyloysl residues from the non-reducing end of XyG cell wall components and XyG oligosaccharides (Frankova *et al.*, 2013; Pauly *et al.*, 2016; B. Zhang *et al.*, 2021). Interestingly, while XyG-deficient *A. thaliana xxt* mutants exhibit only minor morphological phenotype changes, *xyl1* mutants lacking α-xylosidase enzyme activity exhibit altered XyG side chains, free XyG oligosaccharide accumulation, and specific phenotypic defects during reproduction, seed dispersal, germination, and seedling growth. Di Marzo *et al.* (2022) demonstrate that the expression of the *XYL1* gene is directly regulated in developing seeds and fruits by the STK transcription factor.

Box 1. **Xyloglucan remodelling and cell wall biomechanics during *Arabidopsis thaliana* seed and fruit biology**

Specific XyG remodelling by a battery of enzymes (A) has profound roles during reproduction, seed dispersal, and germination (B–E). The control of reproduction by the MADS-box transcription factor STK is achieved in part by αXYL-mediated cell wall remodelling (B) combined with other pathways which may differ between seed and fruit development (see cited references and figure 7 in Di Marzo *et al.*, 2022). The control of silique growth (C) by STK, for example, requires XYL1 with a reduced silique size and increased valve cell wall stiffness in both the *stk* and the *xyl1* mutant. There were no obvious morphological phenotype changes observed in *axy8* and *bglc1* mutants. In contrast to this, *bgal* and *xyl1* mutants exhibited specific seed- and fruit-associated phenotype changes. As for the *xyl1* mutant, reduced silique elongation growth was also observed in the *bgal10* mutant (Sampedro *et al.*, 2012); however, in contrast to the non-dormant *xyl1* mutant seeds, the seeds of *bgal10* mutants are dormant. The seeds of *bgal6* (*mum2*) (Dean *et al.*, 2007), *stk* (Ezquer et al., 2016), and *stk/xyl1* mutants are impaired in mucilage production (B), whereas *xyl1* mutant seeds have wild-type (WT) phenotype and produce mucilage (Di Marzo *et al.*, 2022). As in the *xyl1* mutant, increased cell wall stiffness (C) was also observed in developing seeds of the *stk* mutant (Ezquer et al., 2016) and may lead to its smaller seed size as well as the defects in seed coat development in that *stk*, but not *xyl1*, mutant seeds are impaired in mucilage production (B) and impaired seed abscission [D; from Balanza *et al.* (2016) with permission (https://doi.org/10.1242/dev.135202)] required for seed dispersal (Balanza *et al.*, 2016). STK seems to achieve this via the *MUM2* gene encoding a βGAL6 involved in pectin and possibly also XyG remodelling (Dean *et al.*, 2007; Ezquer et al., 2016). The *bgal10* mutant is also reduced in silique growth (C), impaired in seed mucilage production, and XyG remodelling (Sampedro *et al.*, 2012). The production of dormant seeds (E) is not affected in the *bgal10* and *axy8* (the *AXY8* gene encodes an αFUC) mutants, but *xyl1* mutant seeds are non-dormant (Sechet *et al.*, 2016). Interestingly, the non-dormant *xyl1* mutant seeds are thermoinhibition resistant (E) and have increased hypocotyl cell wall stiffness in creep-extension analysis (Shigeyama *et al.*, 2016). Altered XyG in cell walls and the accumulation of free XyG oligosaccharides (C, E) were associated with the altered fruit and seed phenotypes of the *xyl1* (Iglesias *et al.*, 2006; Sampedro *et al.*, 2010; Günl and Pauly, 2011; Sechet *et al.*, 2016; Shigeyama *et al.*, 2016; Di Marzo *et al.*, 2022), *bgal10* (Sampedro *et al.*, 2012), *axy8* (Günl *et al.*, 2011), and *bglc1* (Sampedro *et al.*, 2017) mutants. DAP, days after pollination.

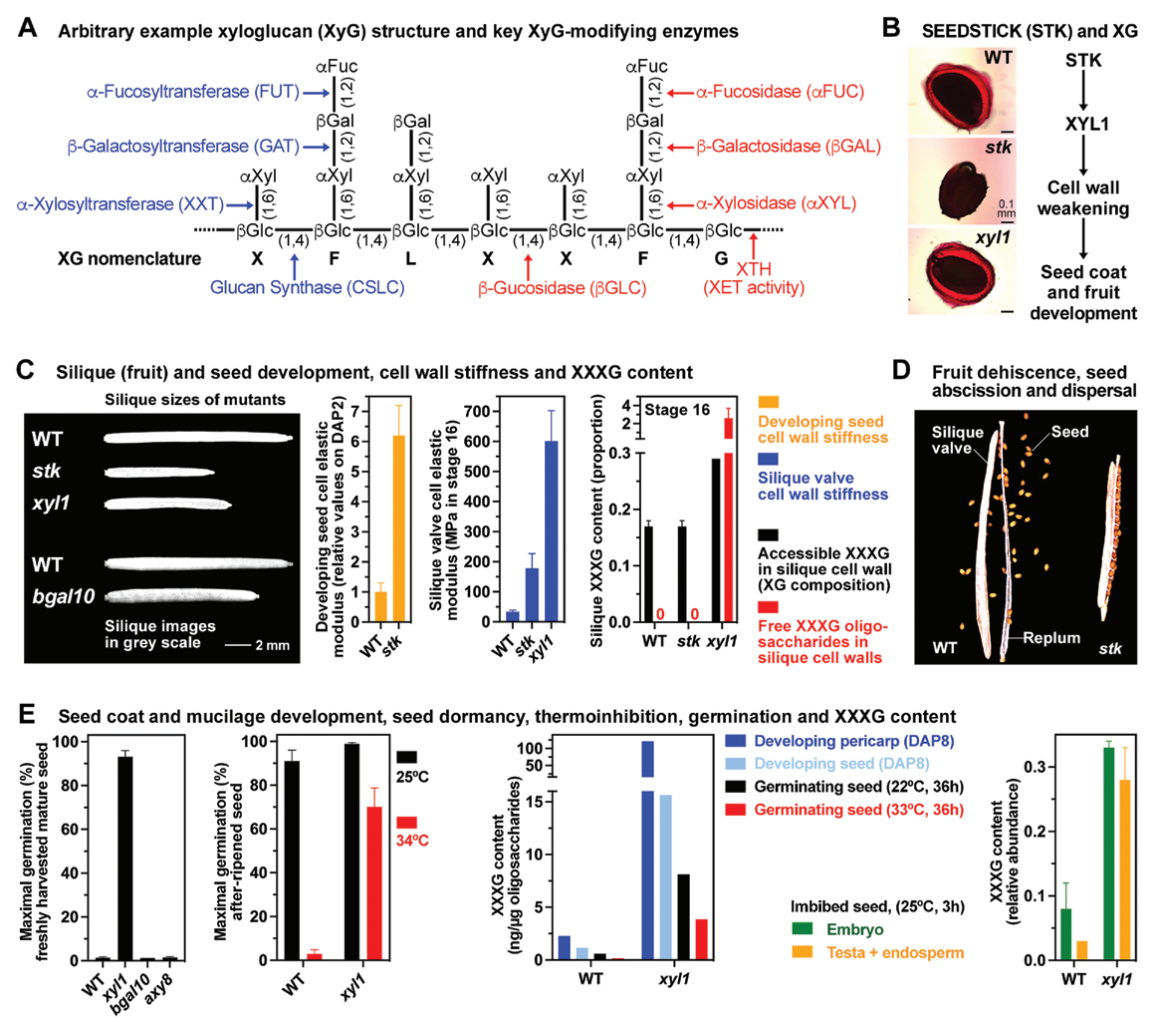

Box 1 summarizes seed- and fruit-associated morphological, biochemical, biomechanical, and physiological changes of *xyl1* and *stk* mutants, including reduced silique elongation growth and increased cell wall stiffness in both, as well as altered XyG side chains, accumulation of free XXXG oligosaccharides, lack of seed dormancy, and increased seed thermotolerance of the *xyl1* mutant (Sampedro *et al.*, 2010; Günl *et al.*, 2011; Sechet *et al.*, 2016; Shigeyama *et al.*, 2016; Di Marzo *et al.*, 2022). Likewise, results from *bgal10*, *bgal6 (mum2*), *axy8*, and *bglc1* mutants are presented which have reduced β-galactosidase, α-fucosidase, and β-glucosidase enzyme activities, respectively. They all have cell wall XyG with altered side chains and free XyG oligosaccharide accumulation (Iglesias *et al.*, 2006; Dean *et al.*, 2007; Günl *et al.*, 2011; Sampedro *et al*., 2012, 2017). *XYL1* and the transcriptional regulation of its expression by STK plays a major role in the control of seed and fruit mechanical properties by XyG remodelling (Box 1); however, depending on the specific process or tissue, other interacting pathways may dominate.

An integrated approach combining genetics with biomechanical and image analysis appears to be important for advancing our understanding of XyG remodelling and cell wall mechanics in seed and fruit biology (Sechet *et al.*, 2016; Shigeyama *et al.*, 2016; Di Marzo *et al.*, 2022). Using atomic force microscopy (AFM) to analyse silique valve cell wall stiffness, Di Marzo *et al.* (2022) demonstrate that developmentally regulated *XYL1* gene expression is required for maintaining wall integrity during silique growth. Using creep-extension analysis with elongating stem segments, Shigeyama *et al.* (2016) reported that *xyl1* mutant cell wall stiffness was higher than in wild-type plants. This work also demonstrated that epidermal cells of *xyl1* mutant siliques are longitudinally shorter and horizontally enlarged, a finding which fits with the increased cell wall stiffness in *xyl1* mutant siliques reported by Di Marzo *et al.* (2022). Although different biomechanical methods were used, in both cases the same conclusion about the role of αXYL in controlling cell wall mechanical properties (stiffness) was obtained. Interestingly, the silique elongation growth is reduced in XyG-deficient *xxt1/xxt2* mutants (Sechet *et al.*, 2016), and the cell wall stiffness tested by microtensile assays of hypocotyls was also decreased compared with the wild type (Cavalier *et al*., 2008). The importance of the right balance in XyG remodelling enzymes (Box 1) seems crucial, and both XXT-mediated incorporation and αXYL-mediated removal of xylosyl residues can lead to the same biomechanical changes.

The αXYL-catalysed cleavage of xylosyl residues from the non-reducing ends of cell wall XyG chains and XyG oligosaccharides has been shown to be the limiting step in XyG oligosaccharide degradation (Iglesias *et al.*, 2006; Shigeyama *et al.*, 2016; Sampedro *et al.*, 2017). Released XyG oligosaccharides can also alter cell wall properties by incorporation catalysed by XyG endotransglycosylase (XET) enzyme activity (Box 1). In grass caryopses, this may lead to coleorhiza-enforced dormancy due to tissue stiffening (Holloway *et al.*, 2021) and in tomato and other endospermic seeds tissue to weakening of the micropylar endosperm (Finch-Savage *et al.*, 2006; Steinbrecher *et al.*, 2017). XyG oligosaccharides were also proposed to directly or indirectly mediate cell wall signalling which can result in altered hormonal biosynthesis or signalling (Frankova *et al.*, 2013; Pauly *et al.*, 2016; Sechet *et al.*, 2016; Shigeyama *et al.*, 2016; B. Zhang *et al.*, 2021). The structure of XyG differs between plant species especially in diversity of the side chains; however, despite this, conservation in XyG remodelling mechanisms and enzymes was also established (Pauly *et al.*, 2016; Rubianes *et al.*, 2019; Holloway *et al.*, 2021). Mutants in XyG remodelling enzymes, such as in STK and XYL1 in the work of Di Marzo *et al.* (2022), are indeed highly suited to advance our understanding of the mechanisms of cell wall biochemistry and biomechanics (Box 1).

Within the Brassicaceae, the dimorphic diaspores of *Aethionema arabicum* offer another interesting approach into cell wall biology during reproduction (Box 2). In *Ae. arabicum*, the developmental control and plasticity of fruit and seed morphs is associated with morphological, biomechanical, gene expression, and physiological differences between the morphs (Lenser *et al*., 2016; Wilhelmsson *et al.*, 2019; Arshad *et al*., 2029, 2021). Comparing the distinct seed and fruit morphs of heteromorphic species therefore provides very interesting systems for future research into cell wall biochemistry and biomechanics including for XyG remodelling enzymes (Box 2).

Box 2. **Biomechanics and XyG remodelling enzymes during *Aethionema arabicum* fruit and seed dimorphism**

Heteromorphic species can produce seed and fruit morphs that are distinct in dispersal, germination, morphology, and physical properties (Lenser *et al*., 2016). The dimorphic species *Aethionema arabicum* naturally exhibits the production of two different seed and fruit morphs on the same plant (A). In addition to this interesting developmental control, it exhibits phenotypic plasticity in that the ratios and numbers are controlled by environmental cues during reproduction. Comparative transcriptome analysis of the dimorphic fruit and seed developmental programme revealed differences in transcription factor and downstream gene expression (Wilhelmsson *et al.*, 2019; Arshad *et al.*, 2021). This includes the transcript abundances of STK and XyG remodelling enzymes (B), and suggests that XyG may differ between the fruits and seed coats of the two morphs. *Aethionema arabicum* develops a larger dehiscent fruit (DEH) with 2–4 M^+^ seeds (with mucilage) and a smaller indehiscent fruit (IND) with a single non-mucilaginous (M^–^) seed (A). Fruit opening in IND fruits needs significantly higher forces than in DEH fruits. When the linear regions of individual force displacement curves (the part prior to breakage) are compared (C), IND fruits (separation area 0.86±0.03mm^2^) show a faster increase in force per mm and therefore a higher elastic modulus than DEH fruits (separation area 6.94±0.14mm^2^) (Arshad *et al.*, 2019). Dimorphic fruits with distinct cell wall architecture are ideal model systems to investigate the effects of cell wall polysaccharide composition and dynamics on seed and fruit size, as well as their biomechanical properties and developmental patterns.

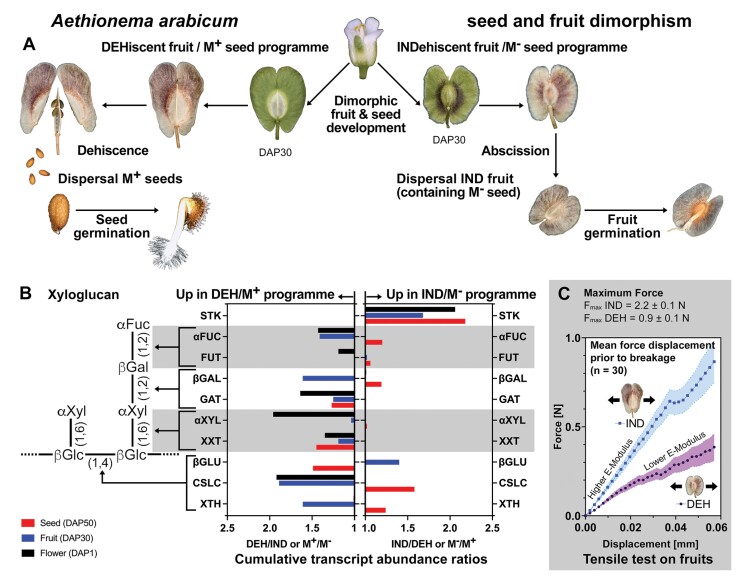

Environmental conditions play a key role in seed and fruit biology (Finch-Savage *et al.*, 2017; Fernandez-Pascual *et al.*, 2019). Temperature during reproduction can shift the ratios and numbers of the *Ae. arabicum* fruit and seed morphs (Lenser *et al*., 2016). Temperature and photoperiod contribute to population fitness by affecting seed coat cell wall properties (thickness, proanthocyanidin content) and thereby dormancy in other species (Finch-Savage *et al.*, 2006; Mizzotti *et al.*, 2014; MacGregor *et al.*, 2015; Fernández Farnocchia *et al.*, 2021). The cell wall is a highly dynamic and adjustable structure, and its biomechanical properties are determined by specific cell wall compositions for which new modelling approaches are being pursued (B. Zhang *et al.*, 2021; Y, Zhang *et al.*, 2021). Integrating molecular work with morphological and biomechanical analysis, as exemplified by Di Marzo *et al.* (2022), and further with such novel modelling approaches are promising prospects for future research into this fascinating topic.
